# Promoting effective child development practices in the first year of life: does timing make a difference?

**DOI:** 10.1186/1471-2431-14-222

**Published:** 2014-09-05

**Authors:** Anna Roia, Elena Paviotti, Valentina Ferluga, Marcella Montico, Lorenzo Monasta, Luca Ronfani, Giorgio Tamburlini

**Affiliations:** Institute for Maternal and Child Health – IRCCS “Burlo Garofolo”, Via dell’Istria 65/1, 34137 Trieste, Italy; Centro per la Salute del Bambino, Trieste, Italy

**Keywords:** Early childhood, Audiovisual materials, Parent–child interaction, Parenting programs, Timing

## Abstract

**Background:**

There is an increasing need for parenting programs aimed at promoting parent–child interaction. A variety of interventions have been proposed. The use of audiovisual materials for parents has been shown to be effective but limited information is available on the optimal timing for its use, particularly for new parents during the first year of life of their children. The aim of this study is to compare the effectiveness of a video administered at two different times to first-time parents in modifying parental knowledge, attitudes and intentions with regards to effective care practices.

**Methods:**

Open randomized controlled trial carried out in a referral mother and child hospital. Eligible parents were randomly assigned to receive a video at one month (early intervention) or at seven months (late intervention) of age of their child. The video addressed four specific activities related to early child development: reading aloud to the baby, early exposure to music, promotion of early socialization for parents and for children. The primary outcome was the proportion of parents who declared that their knowledge, attitudes and intentions changed after having seen the video at one or seven months of age of the child.

**Results:**

One hundred and five families were randomly allocated either to the early (53) or to the late (52) intervention group. For 99 families (52 in the early and 47 in the late group) a complete outcome evaluation was available. Parents included in the early administration group more frequently reported modifications in their knowledge of the suggested practices while parents in the late group more frequently reported a change in their attitudes. This finding was consistent across all four practices. The video was found to influence parental intentions in the great majority of interviewed parents with no significant difference between groups (82.7% and 87.2% in the early and late intervention group, respectively).

**Conclusions:**

Audiovisual materials can be an effective complementary tool in programs aimed at supporting parents, particularly those dealing with their first baby. The results provide some useful insights into the differential benefits of using audiovisual aids at different times during the first year of life of the baby.

**Trial registration:**

ClinicalTrials.gov NCT02120430

**Electronic supplementary material:**

The online version of this article (doi:10.1186/1471-2431-14-222) contains supplementary material, which is available to authorized users.

## Background

Early experiences affect both structure and functioning of the brain and child development. In particular, interactions with caregivers during the first years of life are crucial to ensure an adequate psychosocial development in children [1]. Conversely, lack of stimulation is associated with early social disadvantage [2–4].

A variety of interventions, delivered through home visits, group sessions with caregivers, individual counselling, or combined approaches have been proposed and appear to be effective in promoting parent–child interaction [1, 5–7]. When associated with active strategies to promote behavioural changes in caregivers, such as feed-back, coaching and role playing, parenting programmes appear to be more effective than when based on information alone [8–10].

Although their usefulness is increasingly recognized, the costs and organizational issues of parenting programmes, particularly when based on planned home visitation, may make their implementation difficult [11–13]. A variety of technological supports have been proposed in order to overcome these obstacles. The use of audiovisual aids for parental counselling has already been evaluated [14–16], and experiences have been described in which the interaction between children and parents was videotaped and discussed with families to promote early child development [8, 17–21]. Evidence on the efficacy of videotapes, DVDs or other electronic media conveying messages aimed at promoting good health practices is available for adults and children [22–25]. However, the choice of the optimal timing for this kind of interventions, while usually based on the critical periods of child development and mother-infant interaction [26], is supported, to our knowledge, by very limited evidence with regard to parental acceptance. This appears to be particularly relevant when targeting first-time parents, who usually receive a lot of new information within a short period of time. Although the optimal balance between the usefulness of a message and its ability to be received by parents may depend on many contextual factors, such as family context, information load and quality, there may be some empirical indications on when such messages could best be delivered. Thus, based on the hypothesis that the effectiveness of information provided to first-time parents in modifying their knowledge, attitudes and intentions regarding rearing practices may depend on when the information is delivered, we designed a study to compare the effectiveness of administering a video at two different times, i.e. at one and seven months of age of the child.

## Methods

The study was designed as an open randomized controlled trial and was carried out at the maternity ward of the Institute for Maternal and Child Health - IRCCS “Burlo Garofolo”, Trieste (Italy), a referral hospital and research centre. The study was conducted in compliance with the Helsinki Declaration and was approved by the Independent Bioethics Committee of the Institute (Prot. CE/V-76, June 11, 2007 and CE/V-86, April 28, 2008), with written informed consent required for enrolment. Inclusion criteria for parents were: first parenting experience, local residence, no major health or psychosocial problems in parents and in the newborn, no delay in discharge from the maternity ward, adequate knowledge of Italian.

Eligible parents were randomly assigned to receive the video:

1. during the first month of life of the child (early intervention group);
2. during the seventh month of life (late intervention group).

The first month of life of the child was chosen since most of the information on parenting is usually provided during this period. The seventh month of the child was chosen because several interventions on parenting (i.e. promotion of reading aloud to children) are devised to start at this age.

The video was delivered through a home visit by a psychologist with specific experience in working with parents.

Randomization was centralized and carried out by the Epidemiology and Biostatistics Unit of the Institute using a computer-based method (Stata/IC version 9, StataCorp 2005, College Station, TX, USA). The allocation concealment was guaranteed by the use of closed opaque envelopes, consecutively numbered. In each envelope a patient’s allocation group was indicated, based on randomization. The researchers opened the first available envelope and assigned the patient to the corresponding group.

### The Video

The video was developed *ad hoc* for the present study by a multidisciplinary team that included clinicians (psychologists, paediatricians) with expertise in parenting support and related programmes, and by a filmmaker. It was conceived as an aid to parenting in the first year of life. It provides an opportunity for parents to look at their own experiences, including the common problems encountered in every day care and child-rearing practices, reflected in the images and in the voices of “normal” caregivers from different socioeconomic backgrounds.

The video lasts 24 minutes and describes “the birth of a new relationship” between primary caregivers (mostly mothers, but also fathers and grandparents) and their babies, in chronological order, starting from late pregnancy to the end of the first year. Caregiver-child interactions are shown from early contact after delivery to common care practices, such as breastfeeding and feeding, vocal exchanges and play, with an emphasis on four specific activities, which are believed to improve child development and caregiver-child interaction: reading aloud to the baby, exposure to songs, rhymes and music, promotion of early socialization for parents and for children [27–31]. All the proposed sequences are meant to represent positive interactions, but no open recommendations to parents are given. Only the voices of the caregivers and the babies are audible, the intention being that of representing real life situations rather than showcasing ideal situations.

### Outcomes

The primary outcome of our study was the proportion of parents who, after being exposed to the video in one of the two different periods, declared that their knowledge, attitudes and intentions regarding any of the four specific activities examined (reading aloud, exposure to music, caregiver and child early socialization) had changed. Details on how the video was used and on the feelings it elicited were also collected.

Information was collected through a researcher-administered structured questionnaire, which includes two sections: section A collected details on the demographic and socio-economic characteristics of the family and on pregnancy, delivery and immediate post-partum; section B focused on how the video was perceived, and on whether it prompted any modification in knowledge, attitudes and intentions. The Additional file 1 provides details on the main issues addressed by the questionnaire and on the type of questions asked. The questions related to the study outcomes are described extensively. Most of the questions, and all of those used for the present analysis, were closed-ended. Section B used Hamblin’s approach (1974) [32], which identifies three levels of evaluation: reactions, learning and behaviours. Furthermore, to assess changes in parental competence, section B was based on Bloom’s theory (1986) [33] which distinguishes between knowledge, skills and attitudes, limiting questions to the first and last fields, since there were no practical abilities to be assessed, and focusing on reading aloud, exposure to music and early socialization.

To explore the feelings elicited by the video we used eight categories of emotions, as proposed by Colasanti and Mastromarino (1994) [34]: happiness, sadness, fear, rage, guilt, sense of competence, lack of self-confidence, and sense of inadequacy.

Interviews were administered by a trained psychologist, unblinded to the purpose of the study and to the allocation group, in the course of a home visit carried out on average two weeks after the delivery of the video.

### Statistical analysis

Categorical data are presented as numbers and percentages, continuous data as means and standard deviations. Differences between groups were evaluated with a chi-square test (or a Fisher exact test when appropriate) for categorical variables and with the Mann–Whitney test for continuous variables, since a non-normal distribution of data was found both with the Kolmogorov–Smirnov test and with normal probability plots. A multivariate logistic regression analysis was carried out to control for differences between the two groups at enrolment and to control for any effect of socio-demographic variables on the association between outcome measures (knowledge, attitudes and intentions) and early and late intervention. Analyses were carried out with the Stata/IC version 9 for Windows (StataCorp 2005, College Station, TX, USA).

## Results

We contacted a convenience sample of 127 families living in the Trieste area, immediately after birth, while mother and baby were still admitted at the maternity ward. Twenty-two families (17.3%) refused to participate, while 105 families were randomly assigned to the early (53) or to the late intervention group (52). Ninety-nine families (52 in the early and 47 in the late group) completed the study, while six families were lost to follow up (5.7%).

There were no differences between the two groups in terms of main socio-demographic characteristics, except for maternal formal education level which was higher in the late intervention group (p = 0.03) (Table 1).

**Table 1:** **Socio-demographic characteristics of the enrolled population**

	Intervention at 1st month (early group, n = 53)	Intervention at 7th month (late group, n = 52)	p
**Sex of the infant: female, number** (%)	22 (41.5)	24 (46.1)	0.6
**Mother’s age in years, mean (SD)**	33.4 (0.7)	33.8 (0.6)	0.7
**Mother’s nationality: Italian, number** (%)	47 (88.7)	44 (84.6)	0.5
**Educational level of the mother**, **number** (%)			0.03
Completed primary or secondary school	11 (20.7)	2 (3.8)	
Completed high school	22 (41.5)	26 (50.0)	
Bachelor degree or higher	20 (37.7)	24 (46.1)	
**Employed mother, number** (%)	42 (79.2)	47 (90.4)	0.1
**Mother in maternity leave, number** (%)*	42 (100.0)	43 (91.5)	0.1^#^
Father’s age in years, mean (SD)**	35.7 (5.8)	37.0 (6.9)	0.4
**Father’s nationality: Italian, number** (%)	44 (86.3)	45 (88.2)	0.8
**Educational level of the father, number** (%)			0.6
Completed primary or secondary school	13 (25.5)	9 (17.6)	
Completed high school	24 (47.1)	25 (49.0)	
Bachelor degree or higher	14 (27.4)	17 (33.3)	
**Father employed, number** (%)	49 (96.1)	49 (96.1)	1.0^#^
**House ownership, number** (%)	43 (81.1)	41 (78.8)	0.8
**Extended family** ^**§**^ **, number** (%)	1 (1.9)	2 (3.8)	0.6^#^

*only for employed mothers (n = 42 and 47).

**Father’s data available for 51 subjects in both groups.

^§^defines a family with extension beyond the nuclear family, including grandparents, aunts, uncles.

^#^Fisher exact test.

### Knowledge, attitudes and intentions

The results concerning the effect of the video on knowledge and attitudes are shown in Table 2.

**Table 2:** **Effect of the video on knowledge**, **attitudes and intentions in the early and late intervention group**

	Intervention at 1st month (early group, n = 52)	Intervention at 7th month (late group, n = 47)	p
**Acquisition of new knowledge regarding**			
Start reading aloud early, number (%)	31 (59.6)	11 (23.4)	<0.001
Start playing music early, number (%)	22 (42.3)	17 (36.2)	0.5
Parental socialization, number (%)	3 (5.8)	1 (2.1)	0.6
Infant socialization, number (%)	7 (13.5)	0	0.01^#^
**Acquisition of positive attitudes towards**			
Start reading aloud early, number (%)	5 (9.6)	15 (31.9)	<0.01
Start playing music early, number (%)	4 (7.7)	17 (36.2)	<0.001
Parental socialization, number (%)	1 (1.9)	7 (14.9)	0.03^#^
Infant socialization, number (%)	3 (5.8)	5 (10.6)	0.5^#^
**Influence on parental intentions, “Yes”, number** (%)	43 (82.7)	41 (87.2)	0.5
If yes in which field:			
Start reading aloud early	27 (62.8)	26 (63.4)	0.95
Start playing music early	25 (58.1)	27 (65.9)	0.5
Parental socialization	3 (7.0)	7 (17.1)	0.2^#^
Infant socialization	6 (14.0)	7 (17.1)	0.7

^#^Fisher exact test.

Parents included in the early administration group more frequently reported a gain in knowledge while parents in the late group more frequently reported a change in their attitudes. In particular, a statistically significant difference between groups in favor of the early group was found for the acquisition of new knowledge regarding the importance of early reading aloud (p < 0.001) and of infant socialization (p = 0.01, Fisher exact test). Conversely, a significant difference between groups in favor of the late intervention group was found for the acquisition of positive attitudes towards early reading aloud (p < 0.01), early exposure to music (p < 0.001) and parental socialization (p = 0.03, Fisher exact test). The video was perceived by the majority of the interviewed as effective in modifying their intentions (82.7% in the early intervention group vs. 87.2% in the late intervention group, p = 0.5), especially with regards to reading aloud and exposure to music, but with no statistically significant difference between the groups (Table 2).

The study outcomes were not affected by either maternal education or by any other socio-demographic factor at multivariate analysis.

### Assessment of how the video was used and on feelings elicited

The DVD was watched mainly by both parents together (59.6% in the early group and 57.4% in the late group, p = 0.8). The situations described in the video were judged as very realistic by 81% of the parents interviewed in both group. Seventy-three percent in the early group and 63.8% in the late group thought the length of the video was adequate (p = 0.3), while it was too short for 26.9% and 29.8% of the respondents, respectively. Only three subjects in the late group (6.4%) defined the video as too long. Parents liked the video much or fairly in 100% of cases in the early group vs. 91.5% in the late group (p = 0.05, Fisher exact test), and the contents were judged to be useful (very or somewhat) by 88.5% and 78.7% of the interviewed respectively (p = 0.2). Home visits were well accepted by parents and 100% of them in the early and 93.6% in the late group thought they had been useful (p = 0.1, Fisher exact test). Thirty-six percent of parents watched the video more than once in both groups (p = 1.0). Consequently, no statistically significant difference was found between groups for all of the above mentioned answers.

**Figure 1:**
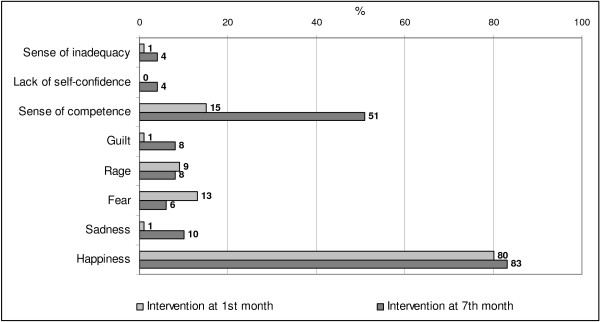
**Feelings elicited by the video.**

Figure 1 describes the feelings elicited by the vision of the video. Parents in the late group felt more frequently a sense of competence compared to parents in early group (p < 0.001) while no difference was found between the two groups for what concerns the other feelings.

Parents provided some suggestions on the best way to watch the video, the main ones being to watch the video also before birth and to watch it together with other family members and other parents, and possibly with the support of a professional.

## Discussion

Our study was aimed at assessing the optimal timing of administration to first-time parents of a video addressing practices that may positively influence child development and parent–child interactions. To our knowledge, there are no other studies exploring the optimal timing of administration of visual aids in parenting programs during the first year of life. We acknowledge that our study cannot fully address the many factors that may influence parental acceptance of a video, let alone their knowledge, attitudes and intentions. Most importantly, our findings rely on what parents report, and we were not able to assess change in actual parenting practices. However, we were able to evaluate changes in parental knowledge, attitudes and intentions, and we can reasonably assume, based on behavioural change theories [35] that modifications in these domains can influence parental practices later on.

Our findings suggest that the video has greater effect on parental knowledge when administered within the first month of age of the baby, and on attitudes when administered at seven months. The most likely explanation for this finding is that parents between the first and the seventh months are exposed anyway, to some extent, to information on these practices, which are becoming increasingly popular in Italy [36], and that the video in fact provides extra support to parental attitudes when seen at a later stage, in close correspondence to the period when such practices are expected to start. This concerns early reading and music in particular, but also early socialization between infants and their parents, which is likely to be perceived as important only after the first months of the baby’s life, when parents’ attention is more focused on primary needs such as feeding. In any case, the video seems to modify intentions in the majority of the parents irrespective of the time.

The fact that parents in the late administration group felt more frequently a sense of competence after looking at the video is not surprising since by the seventh month most of them would have overcome the feelings of lack of self-confidence which are common among parents during the first month of life of their first baby.

## Conclusions

Our results support the idea that audiovisual materials, if properly designed and administered, can be an effective complementary tool in programs aimed at supporting parents, particularly when dealing with their first baby [8, 9, 19]. They also provide useful insight about the differential benefits of using such visual aids at different times during the first year of the baby. In this respect, the importance of an appropriate setting of administration, ideally through a home visit as in our study, cannot be overlooked, as it allows for sufficient time to introduce the video and its purpose [9, 10, 25, 37] and contribute to making parents feel more at ease. This aspect may be even more important when dealing with population groups which, due to specific cultural or social reasons, are more difficult to reach out to and yet are those that would yield the greatest benefit from such interventions [36, 38–40].

## Electronic supplementary material

Additional file 1: **Researcher-administered structured questionnaire: main issues addressed and type of questions asked.** (DOC 50 KB)
