# Supplementary material for: Promoting effective child development practices in the first year of life: does timing make a difference?
Source: BMC Pediatr. 2014 Sep 5;14:222. doi: 10.1186/1471-2431-14-222 (PMC4177066; doi:10.1186/1471-2431-14-222)
Supplement: Supplementary file 1 — Additional file 1: Researcher-administered structured questionnaire: main issues addressed and type of questions asked. (DOC 50 KB) [file 12887_2013_1158_MOESM1_ESM.doc]

**Researcher-administered structured questionnaire: main issues addressed and type of questions asked**

**Section A**

| **Questions** | **Type of question** |
| --- | --- |
| **Demographic data and social background** (for both parents)   - age - citizenship - years of residence in the study area - formal educational level - employment | Number  Multiple choice  Number  Multiple choice  Multiple choice |
| **Information about the family**   - home ownership - number of children - number of pregnancy - extended family | Yes/No  Number  Number  Multiple choice |
| **Pregnancy**   - pregnancy problems - stressful events - drugs use - emotion elicited (maximum 3 allowed among happiness, sadness, fear, rage, guilt, sense of competence, lack of self-confidence, and sense of inadequacy) - social support (by health personnel/by family members) | Yes/No*  Yes/No*  Yes/No*  Multiple choice  Yes/No |
| **Delivery**   - problems - emotion elicited (maximum 3 allowed among happiness, sadness, fear, rage, guilt, sense of competence, lack of self-confidence, and sense of inadequacy) - adequate support (by health personnel/by family members) - cesarean section | Yes/No*  Multiple choice  Yes/No  Yes/No |
| **Immediate post-partum**   - immediate post-partum problems - emotion elicited (maximum 3 allowed among happiness, sadness, fear, rage, guilt, sense of competence, lack of self-confidence, and sense of inadequacy) - social support (by health personnel/by family members) - breastfeeding - problems concerning breastfeeding - need of support for breastfeeding | Yes/No*  Multiple choice  Yes/No  Yes/No**  Yes/No  Yes/No |
| **Stressful events from the first to the seventh month of the baby’s life** (concerning the mother, the father or the infant) | Free text |

* If the answer was “Yes”, the respondent was asked to specify in an open field.

** If the answer was “Yes”, the respondent was asked to specify whether breastfeeding was exclusive/predominant or complementary.

**Section B**

| **Informations on the use of the video**   - number of times the video has been viewed (one time; more than one times) - who viewed the video (only the respondent; respondent with partner; respondent with other person) | Multiple choice  Multiple choice |
| --- | --- |
| **Video acceptance (study outcome)**   - duration (too long; too short; adequate) - realism of the situations proposed (very realistic, fairly realistic, unrealistic) - video global acceptance (much liked, fairly, did not like) - emotions elicited by the vision (maximum 3 allowed among happiness, sadness, fear, rage, guilt, sense of competence, lack of self-confidence, and sense of inadequacy) - Most positive video aspects - Most negative video aspects | Multiple choice  Multiple choice  Multiple choice  Multiple choice  Free text  Free text |
| **Learning and behavior (study outcome)**:   - usefulness of the video on parenting (very, somewhat, not useful) - changes in parental competence after the vision (see below) - changes in parental behavior after the vision (see below) | Yes/No*  Multiple choice  Multiple choice |

* If the answer was “Yes”, the respondent was asked to specify in an open field

After watching the video, in which of these areas you believe there have been changes (acquisition of new knowledge and attitudes) on parenting?

|  | **Knowledge** | **Attitudes** |  |
| --- | --- | --- | --- |
| Child feeding |  |  |  |
| Relationship with the baby |  |  |  |
| Relationship with the partner |  |  |  |
| Request for social supports |  |  |  |
| Sharing emotions with familiar people |  |  |  |
| Sharing emotions with health workers |  |  |  |
| Early start reading aloud |  |  |  |
| Early approach to music |  |  |  |
| Socialization among parents |  |  |  |
| Socialization among infants |  |  |  |
| None of these |  |  |  |
| Other (free text) ______________________________________________________ | | | |

In your opinion, did the video influence your future behaviors as a parent?

Yes/No

If yes, in which areas?

- Child feeding

- Relationship with the baby

- Relationship with the partner

- Request for social supports

- Sharing emotions with familiar people

- Sharing emotions with health workers

- Early start reading aloud

- Early approach to music

- Socialization among parents

- Socialization among infants

- None of these

- Other (free text)
